# Evolution of Culture on Patient Safety in the Clinical Setting of a Spanish Mutual Insurance Company: Observational Study between 2009 and 2017 Based on AHRQ Survey

**DOI:** 10.3390/ijerph18189437

**Published:** 2021-09-07

**Authors:** Miguel Ángel Ulibarrena, Leire Sainz de Vicuña, Ignacio García-Alonso, Pablo Lledo, Marta Gutiérrez, Asier Ulibarrena-García, Víctor Echenagusia, Borja Herrero de la Parte

**Affiliations:** 1 Department of Surgery and Radiology and Physical Medicine, Faculty of Medicine and Nursing, University of the Basque Country UPV/EHU, 48940 Leioa, Spain; mulibar@mutualia.eus; 2 Mutualia, 48009 Bilbao, Spain; lsainz@mutualia.eus (L.S.d.V.); plledo@mutualia.eus (P.L.); mgutierrez@mutualia.eus (M.G.); uliasi@outlook.es (A.U.-G.); vetxena@mutualia.es (V.E.); 3 Department of Surgery and Radiology and Physical Medicine, University of the Basque Country, 48940 Leioa, Spain; ignacio.galonso@ehu.eus; 4 Interventional Radiology Research Group, Biocruces Bizkaia Health Research Institute, 48903 Barakaldo, Spain

**Keywords:** patient safety, AHRQ survey, patient safety culture

## Abstract

Background: Patient safety (PS) is a key factor in reducing or even eradicating adverse incidents and events. Many health organizations promote strategies to improve PS, while also pointing out the importance of measuring it. For more than eight years, our institution has developed strategies focused on improving PS-culture among our personnel. The goal of this paper is to analyze the PS-culture between the years 2009 and 2017. Methods: A cross-sectional survey focused on PS, and developed by the American Agency for Healthcare Research and Quality (AHRQ), was conducted in 2009 and in 2017 among all healthcare workers at Mutualia, anonymously and voluntarily. Results: The overall response rate was similar in both 2009 and 2017 (37.2% and 38.5%, respectively). The average rating obtained showed a significant improvement over the period (7.7 vs. 8.1; *p* < 0.05). Itemizing by question, the main strengths were found in management support, organizational learning and continuous improvement, and, especially, in teamwork. Regarding weaknesses, the two lowest scores were those which refer to the balance between clinical safety and workload and the freedom to question the decisions made by superiors. Conclusions: The results obtained from the PS-surveys show that the overall PS-culture in our institution has increased, suggesting that the strategies focused on the improvement of PS-culture were well adopted among our personnel. The overall score places Mutualia at similar levels to those reached by the AHRQ and Spanish National Health System.

## 1. Introduction

One of the current challenges in medicine is providing safe health care in environments that are ever more complex, technological, and in which more and different professionals participate in ever-changing situations and almost always under pressure. In these situations, it is not infrequent for problems to appear and patients to be hurt.

Though adverse effects reported in primary care services in Spain amount to 1.1% [1], the figure rises to 10% when focusing on in-patients [2,3]. This is a major health problem and a huge management challenge for all health systems. More so, if we consider that a report from the Ministry of Health and Consumption indicates that around 40–70% of these events could be prevented.

The American Agency for Healthcare Research and Quality (AHRQ) defines the culture of patient safety (PS) as: an individual and organizational pattern of behavior based on shared beliefs and values that continuously seek to minimize harm to the patient that might arise from healthcare processes. The culture of the PS among health professionals is a key factor in keeping adverse incidents and events from arising [4,5]. In fact, spreading the culture of clinical safety among medical students in one of the main strategies of the World Health Organization (WHO) [6]. Moreover, the National Quality Forum in the United States highlights this as the first practice to improve PS, while also pointing out the importance of measuring it [7]. This aspect is also a priority for the Spanish National Health System (SNS) in its Quality Plan where, since 2005, the Patient Safety strategy has been laid out, and was recently updated for the five-year period 2015–2020 [8].

Our insurance company, Mutualia, is a Spanish health insurer of work-related accidents and occupational diseases, which collaborates with the Spanish Social Security System, number 2 (MCSS-2), headquartered in the Basque Autonomous Community. Providing medical care due to occupational accidents or illnesses is one of its functions as a mutual insurance company. It cares for approximately 400,000 protected workers with approximately 42,000 new patients per year, with medical care that ranges from initial or emergency care to primary health care, rehabilitation, as well as hospitalization and surgical care. Every year, we handle up to 160,000 medical consultations which result in around 2400 admissions and 2000 surgical procedures (most of them within the orthopedic area). To carry this out, Mutualia has a day hospital and two traditional hospitals, with a staff of 600 workers, of whom almost 400 are healthcare workers.

Mutualia agrees with the priorities of national and international organizations in the field of PS, and started working on it in 2008 when the first Mutualia’s Patient Safety Plan (PSP) was drawn up, and the Clinical Risk Management Committee (CRMC) was set up. The CRMC was created to lead the implementation of the PSP, spreading the culture of clinical PS, and also carrying out, collecting and channeling proposals to improve it. 

**Figure 1:**
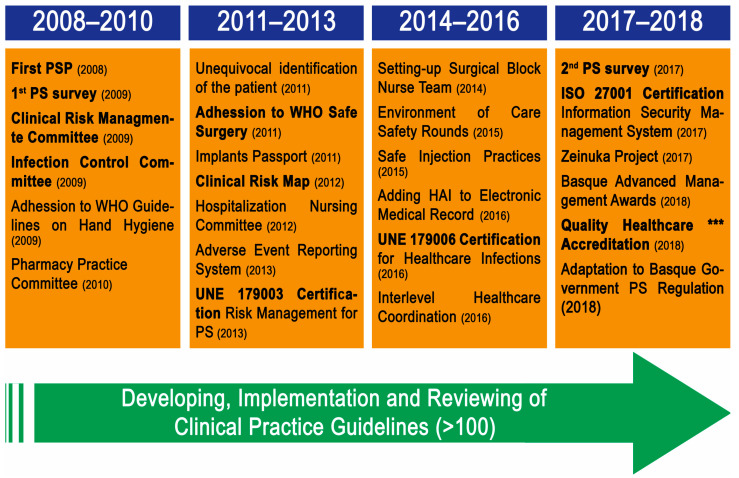
Timeline of the implementation of strategies promoting Patient Safety (PS) at Mutualia.

Since 2008, Mutualia has been working hard, developing strategies to establish and improve PS-culture which have led to several actions (Figure 1).

The first advances in PS were outlined in Mutualia’s Biannual Strategic Plan (2008–2010), where the objectives and actions related to SP were defined, and which were later developed in the first PSP (2008). This plan served as an internal reflection on the PS-culture situation in our organization, a study of the international trends and recommendations on PS, and the selection of the most appropriate PS practices for Mutualia. This work was carried out in a collaborative environment by a team of integrated personnel from different health care areas and medical specialties, with the support of the Department of Organization and Quality. The aim of this first PSP was to provide better quality care by minimizing the risks associated with the health care provided to our patients; relying on the involvement of the organization, its professionals, patients and their environment. For this purpose, we had three main strategies: (a) to promote culture, training and research in SP; (b) to deepen the integration of SP in healthcare activity; and (c) to promote the participation of patients and their environment. The CRMC had the task of leading the implementation of this PSP, by transmitting the PS-culture, making, receiving and communicating proposals for improvement, monitoring the action plan and its indicators on a monthly basis, and adjusting the planning based on the monitoring. Similarly, the Infection Control Committee had the role of minimizing healthcare-related adverse effects related to infections. In 2011, following WHO recommendations, the surgical checklist was implemented in all surgical procedures, adhering to the WHO Safe Surgery strategy, in order to reduce adverse events in the surgical environment. A year later, in 2012, the Patient Safety Risk Map was drawn up and became a very powerful tool for the identification of up to a total of 223 potential risks that patients may suffer during healthcare, 73 of them classified as high or extreme.

After defining and implementing several patient safety-related actions, one of our major worries was to ensure their correct implementation in all our centers and services. That is why in 2015 we implemented patient safety rounds, a system that seeks to complement internal audits, verifying the correct implementation of the defined measures to guarantee PS (compliance with protocols, identification of barriers defined in the risk map and analysis after notifications were received, etc.) in the different Mutualia centers and clinics. Finally, amongst the activities shown in Figure 1, we would like to point out the implementation of the second PSP (2017). In this second PSP we reinforced and upgraded the national and/or international trends and recommendations on the PS-culture to reinforce our PS strategy.

The aim of this paper is to present a comparative study on the Mutualia hospitals in Spain on the perceived patient safety culture, between the years 2009 and 2017, and whether the culture on safety had changed over time in our company.

## 2. Materials and Methods

Using a questionnaire developed by the AHRQ [9], which has been validated in Spain by the University of Murcia [10], we have twice carried out a survey to assess PS-culture among healthcare workers at Mutualia. Since no data containing nominal, sensitive or personal health information were collected, the IRB of our institution (University of The Basque Country) considered that no ethical-approval was required for this research. The investigation complied with ethics requirements established in the good practices code of our institution.

As shown in Figure 1, Mutualia developed the first PSP in 2008, a year before conducting the first PS-culture survey; from that moment on, PS-culture started to spread among Mutualia employees. The first meetings were organized between Mutualia’s executive staff and the heads of units/hospital services of Mutualia. Each service chief then disseminated the information to the personnel under his/her responsibility, supported by the person in charge of the SP strategy.

The same survey was run at two different times, in 2009 and in 2017. The first one was conducted on paper in 2009, and then repeated in 2017 (when the second PSP was developed), but via internet survey, based on the LimeSurvey platform, a free open-source application to perform web-based surveys. In 2009, a single copy of the paper-based survey was handed personally to each worker by the respective unit/service chief, and the employees were completely free to answer it wherever and whenever they preferred. The employees were sensitized to answer all the questions included in the survey, and avoiding leaving any blanks when returning the survey, in order to ensure that missing data did not create artifacts in the interpretation of the results of the PS-culture at Mutualia. To collect the survey, a pick-up box was available within two weeks of the delivery of the survey. Despite these efforts, if incomplete surveys with missing data were detected, they were removed prior to analysis in accordance to exclusion criteria stated by Hellings et al. [11]; in brief these were: an entire section not completed, fewer than half the items answered, and all items answered the same.

For online surveys conducted in 2017, a link was sent by e-mail to each person, who was provided unique and anonymous access to the survey. To answer this survey, it was necessary to provide a valid email address in order to avoid multiple responses by the same person. To prevent missing data, the online survey was designed so that all answers had to be completed to allow the web form to be submitted.

In both paper and internet surveys, responses were then transferred to an Excel datasheet by one of the authors of this study. For the non-web-based survey, in order to minimize human error in data entry, we adopted the double-entry method with checking for mismatches and out-of-range values, where one person enters the data twice. For the web-based survey, the data obtained from web-based surveys were exported directly to an Excel spreadsheet. Blind analyses of the responses were then carried out by another member of the research team.

The survey provided information about 12 aspects of PS-culture which were explored with 42 questions, measured on a 5-level Likert scale (Table A1 in Appendix A). In addition, we explored other parameters related to the type of work, clinical service or work area, and years of professional experience.

For this study, all the aspects and questions were taken into account, excluding none of them. To analyze the results, we followed the same methodology used by the AHRQ [12]: only when one of the two more favorable answers was chosen, was the response considered to be “positive”. That is, for questions written in a positive sense, the answers “always/almost always” or “agree/very much agree” were considered. On the other hand, for questions written in the negative sense, the answers “disagree/strongly disagree” or “never/rarely” were taken into account. In the same way, to identify strengths and weaknesses, we followed the criteria set out by the AHRQ. A topic was considered to be a strength when it reached ≥75% of positive responses. On the other hand, we considered a topic to be an area needing improvement when it didn’t reach 50% of positive responses.

In order to facilitate the analysis of the questions, and because of the huge number of questions, a three-block division was made. Block A included those dimensions and questions that referred to patient safety in global terms. Blocks B and C included questions relating to patient safety at the level of each work unit or service and the hospital as a whole, respectively.

Qualitative variables were described using percentages. Comparison between the two surveys was performed using a contingency analysis and Chi-square (χ²) test. For quantitative variables a Kolmogorov–Smirnov’s test was used to assess the normality of the data. If normality was confirmed, data were described using the mean and standard deviation (SD); if not, the median and range were used instead. To compare data from the two surveys a t-test (parametric) or Mann–Whitney U test (nonparametric) were used. Differences were considered significant when *p* < 0.05. Prism^®^ 8 (GraphPad Software, San Diego, CA, USA) was used.

## 3. Results

Overall, 282 questionnaires were collected between the two surveyed time periods. None of them were excluded according to the exclusion criteria outlined above.

This, Mutualia’s starting point, was a universe of two small hospitals and 14 assistance centers with a total staff of 349 professionals in diverse healthcare sectors (medicine, nursing, pharmacy, administration, etc.). In 2017, these data underwent slight variations, with the number of assistance centers dropping by one to 13, two small hospitals, and a new day hospital, with an overall 13% increase in staff, rising to 395 healthcare professionals. It is important to consider that, because of the organization of our company, most of the staff are in direct contact with patients, which explains why 96.8% of the participants in the survey stated as much in this regard.

It may be observed that the overall score for PS-culture at Mutualia (over 10, obtained from item number 43), rose slightly by four tenths from 2009 to 2017 (7.7 ± 1.4 vs. 8.1 ± 1.3, respectively). This means that, considered globally, a significant improvement was reached (*p* < 0.05).

There was an increase in the total number of responses obtained between both years, 130 vs. 152, which represents a 17% increase in the number of participants. If we consider the percentage of the staff involved, it was 37.2% in 2009 and 38.5% in 2017 (Table 1). Among the respondents in 2017, at least 43 (30%) were new employees, who could not have completed the 2009 survey. The number of years of professional experience, of work at Mutualia, and in the current work area, as well as the rate of full-time workers are given in detail in Table 1. The responses obtained, itemized by type of job or work area, are laid out in Table 2 and Table 3, respectively.

**Figure 2:**
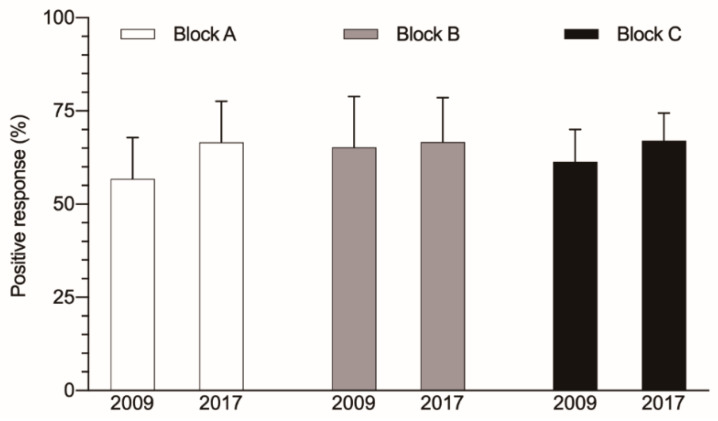
Overall scores for each of the three blocks that comprised the survey. Block A: results of the safety culture (white bar); Block B, aspects of the culture of safety at the unit/service level (grey bar); Block C, aspects of the culture of safety at the whole hospital level (black bar).

The survey is divided into three blocks. The score obtained in each of them is displayed in Figure 2. In Block A, which measures the overall PS-culture, an increase by 10 percentage points can be observed (56.3 ± 11.1% (95% CI: 46.5–67) and 66.8 ± 11.4% (95% CI: 56.1–77.2), in 2009 and 2017, respectively; *p* < 0.05). In Block B, which covers the exploration of PS-culture at the level of the clinical unit or service, only a slight improvement is noted (64.3 ± 13.6% (95% CI: 59.5–71) to 66.9 ± 11.9% (95% CI: 61.7–71.7); *p* = 0.69). In Block C, which measures the safety culture throughout the whole institution, there is also a nonsignificant increase (61.3% ± 8.7% (95% CI: 54.1–68.6) to 67% ± 7.4% (95% CI: 60.8–73.2)).

In general, almost all of the aspects (and each of the questions included in them) improved (Table 4) by a mean of 7.31 ± 3.98 percentage points. The greatest increases were reached in discipline 1 (frequency of reports of events), and discipline 7 (feedback and communication of errors) (*p* < 0.01 and *p* < 0.05, respectively). However, a decrease in 3 disciplines was also recorded: support from direct boss (discipline 3), teamwork in the unit or service (discipline 5), and sufficient personnel (discipline 9). The score registered in these three disciplines dropped by 4.96 ± 5.1 percentage points.

Regarding weaknesses, while it is true that none of the aspects analyzed registered figures below 50%, there are two with scores abating below 60%: aspect 6 (frankness in communication) dropped from 59.3% to 54.07%, and aspect 9 (sufficient personnel) downgraded from 65% to 54.3% (Table 4). 

Now, if we look independently to each of the questions from each aspect, the best results (strengths) were obtained in questions 9, 22, 23, 30, 35, and 57. However, we can also observe that there are two with a score lower than 50% (weaknesses): number 15 and number 37, which refer to the balance between clinical safety and workload and the freedom to question the decisions made by superiors. In more detail, questions categorized as strengths or weaknesses are stated in Table 5.

## 4. Discussion

When we designed the first PSP at Mutualia in 2008, we focused it on three main core ideas, which have been maintained in later versions: (a) to foster the culture, training, and research in Patient Safety, (b) to promote integration of Patient Safety concern and medical practice, and (c) to foster the participation of patients and those around them in the process. However, the weight of each of these three main ideas has been different along the 8 years.

“Training” was a priority in the first PSP (2008–2009). In fact, during the first few years (2008–2009, 2010–2011), we greatly encouraged the training of our personnel via specific courses, workshops, clinical sessions, etc. so nearly all our staff received basic training on PS. From then on, basic training was offered mainly for new hires, though those who wished to refresh their knowledge were allowed to attend the course. Starting in 2010, quarterly clinical sessions over safety incidents have been held with the teams, in which key safety concepts were also refreshed. In this period, mid- and high-level training (even reaching masters-level) of both interlocutors in clinical safety from each service and the members of the Clinical Safety Committee has been promoted. These people make up the CRMC at Mutualia.

Once basic training was widely spread in our company, actions to foster integration of safety into clinical practice became more prominent in the subsequent biennial PSP (2012–2013, 2014–2015) (Figure 1). In our last PSP (2016–2017) more specific actions oriented in the field of participation of the patient and those around them, have been included, such as “focal groups”, working groups involving patients’ associations, etc.

Prior to the discussion of our findings, we must point out the limitations of our research, which are mainly inherent to the tool used for collecting data. It was developed by the AHRQ as a voluntary and anonymous survey. While this freedom of the participants grants sincerity and interest in the responses, it has an intrinsic bias: the picture obtained reflects only the ideas of those more deeply involved in the subject [13]. Moreover, being anonymous it is not possible to assess the evolution as a matched population, which would offer quite strong information. The absence of responses differentiated by sex, age or the degree of specialization of the respondents is another important aspect to take into account, since there are response biases favoring women, young physicians, and non-specialty fellow members [14,15].

Something that could also influence our results is the fact that the staff at Mutualia remained quite stable through the period of the study. This makes training efforts quite efficient, more than they would be in those institutions with a greater turnover of their employees. In fact, 67% of the respondents in 2009 had over 6 years of experience at Mutualia, and this figure rose to 78.2% in 2017. To put this figure into context, in the survey reported by the AHRQ, only 52% of the respondents had over 6 years of experience at their current institutions [16,17].

There are several other characteristics about our population which strongly defer from other reports on PS. However, they are related to the peculiarities of the clinical activity carried out at Mutualia as a mutual insurance company, as compared to a general hospital in Spain (reported by SNS) and the USA (reported by the AHRQ) [16,17,18,19]

Because of the services provided by Mutualia, administrative workers played an important role in patient care. This is why they have been included in our PS activities and consequently in our surveys. If we look at the reports of SNS and AHRQ, these personnel have been excluded. At Mutualia, most respondents come from Rehabilitation, Emergency, and Orthopedics services (52.3%). On the contrary, only 21.5% of responses reported by SNS come from those services [10,18]. Data from the AHRQ are not fully available.

The high percentage of surveyed workers at Mutualia who refer to having a direct patient interaction (96.8%) cannot be explained by these peculiarities. In the AHRQ report the figure only reaches 78% while in the SNS survey it peaks at 93% (neither of them including administrative personnel).

If we focus on the responses obtained from each of these services in 2017, some differences may be observed. The response rate for Rehabilitation workers is about 5–6 times higher than those reported by the SNS and AHRQ. A similar pattern may be observed relating to the Orthopedics Service, for which responses account for a percentage 2–6 times higher than that reported by the AHRQ (these data are included in the “Surgery” work area) and SNS. The percentage of responses corresponding to workers of the Emergency Service is similar to that of the SNS report, but three times higher than those reported by the AHRQ.

It could be thought that the response rate is too low to allow a relevant analysis (37.2%, 38.5%), but it is similar to the one obtained in the SNS, where the response rate at small hospitals was 39.7% [10,18]. However, it is considerably lower than that obtained at AHRQ (52%) [16,17]. Similar studies reported by other insurance companies in our country show much higher response rates. In 2017, Manzanera et al. reported a 61% response rate [20], 20 points higher than the response rate achieved at Mutualia in the same year. This higher response rate could be explained by a higher response rate from personnel in direct contact with patients; 90% of the responses were from physicians, nurses and physiotherapists. In our case, these groups accounted for 73% of the total number of responses. A longitudinal observational study subsequently published by the same research group revealed similar response rates (67% in 2015 and 63% in 2016) [21]. In this particular case, the survey was addressed to quality coordinators, who were also nurses, physicians or physiotherapists with specific training in PS and who were responsible for introducing and disseminating the PS-culture in their workplace and among their colleagues.

This high response rate shows us that, the more health professionals are aware of SP (due to their closer relationship with patients), the greater the acceptance of the surveys. In the study carried out at Mutualia, 25% of the responses came from administrative staff and technicians who, despite having direct contact with our patients, are less involved with them and therefore may perceive the PS-culture as more distant from their work activity and, consequently, have less adherence to answering the survey.

Unfortunately, we are not able to provide information on the reasons for this low participation among our personnel. Several studies focused on the reasons for nonparticipation in different types of research surveys agree on the main underlying motivations for their opposition [22,23,24]. These include: lack of time to respond to the survey, heavier workload, lack of interest in the research, no understanding about the research, not being adequately informed or not being aware of the possibility of participation, and also concerns around response privacy. These studies also include some tips that may be of interest and should be taken into account in future survey-based research, such as providing employees with adequate information about the opportunity to participate and using a less time-consuming survey.

Analyzing the level of awareness of our staff, we notice that it has increased considerably since 2009 (7.7 vs. 8.1), reaching the same level reported by the AHRQ in 2018 for those hospitals with a similar number of beds (25–49) [16,17]. It should be noted that our figures are above the mean obtained at similar hospitals from SNS [10,18]. These results back up the idea that the policy developed by Mutualia has been effective at increasing/maintaining the awareness and involvement of all personnel; moreover, this makes us feel optimistic about the success of future initiatives in this field. Even though we have reduced the weight of generic training over these years, this reduction has been more than made up for by other factors. Among them, the application of best PS clinical practices have shaped the clinical actions of our professionals, which on the other hand, has been reflected in the indicators of care received and also in the satisfaction of our patients. In this sense, Mutualia has obtained, as a result of this effort, several certifications such as UNE 179003 (2013), UNE 179006 (2016), ISO 27001 and Quality Healthcare *** Accreditation (2018) (as shown in Figure 1) [25,26].

The other pillar that we understand to be related to the increase in our PS-culture is the in situ action of the interlocutors in clinical safety. They are expected to be role models in PS for their colleagues, explaining and sharing new actions that are implemented, clearing up doubts, and above all, participating in improvement teams that analyze the risks of each clinical area and propose preventive measures. This way, there are several permanent teams working on PS, such as the Pharmacy, Infections and Use of Antimicrobials, Hand Hygiene, and Surgery Block Nursing teams, and more recently, the Emergency and X-ray Diagnosis teams.

On the other hand, these are not the only influences our professionals receive, because since 2008, PS has become a generalized trend in healthcare institutions. In fact, it has been integrated into the clinical practice of all healthcare professionals and, also in their culture. However, bibliographic references in our country are scarce [20,21,27].

Nevertheless, there are some fields where Mutualia’s results are considerably lower than those reported by the AHRQ, leaving a margin to design new strategies to improve PS. Many of them are included in Block B: communication openness (59.3% vs. 71%), feedback and communication about error (69.4% vs. 75%), organizational learning/continuous improvement (69.4% vs. 75%), and management support for PS (73.9% vs. 78%) [16,17]. Improvements in these aspects have been required from us by the Spanish Association for Standardization and Certification. To fulfil this requirement, several actions have been started. Regarding “learning and continuous improvement”, the “Educator Project” (based on the Swiss cheese model of safety incidents) is focused on risk analysis and management, and the “Safety Pills” and “Txoko” projects provide, via our intranet, continuous training to all our employees (updated material about SP). Regarding communication, the risk management unit has implemented changes relating to the incident communication process and its transmission to the people in charge.

Although most of the items have shown a positive evolution, the areas of supervisor support, teamwork, and sufficient workers has worsened. The two former ones started with very high scores, but as they still remain among the most highly-rated disciplines, we could interpret that the decrease is just moderate. However, the notable decrease in the perception of sufficient personnel is worrying, since it shows that our personnel believe that the number of healthcare workers is not enough to face the workload, while still providing the patient with the best healthcare.

Regarding the strengths that manifested in our survey, overall, the only aspect where Mutualia obtained greater than a 75% positive response rate is in the fifth: teamwork in the unit or service. Paradoxically, in 2017, the score in this area dropped 1.1% when compared to 2009 (79% vs. 77.9%); however, the people who responded considered that this is an area where clinical safety is tightly monitored.

Finally, it is important to point out that our results cannot or should not be generalized to other health insurance organizations of work-related accidents and occupational diseases, especially because of the particular organizational and human resource characteristics of Mutualia as summarized in Table 1, Table 2 and Table 3 (number of employees, type of health care provided, territory in which it operates, etc.), but above all due to the particular actions in terms of PS-culture that Mutualia has taken on, developed and promoted.

## 5. Conclusions

The analysis of the surveys shows that overall PS-culture has experienced a significant improvement at Mutualia between 2009 and 2017. Although, it is true that aspects such as staffing or freedom to question the decisions made by superiors should be further emphasized to improve PS outcomes. All in all, this comparative study on the Mutualia hospitals, between the years 2009 and 2017, shows that our organization is growing in terms of PS-culture, placing itself on similar levels to world role models. It confirms the good line of work that Mutualia has been carrying out, even though there is still a great deal of room for improvement, especially in some specific areas that our survey has outlined in detail.

## Figures and Tables

**Table 1:** Infrastructure, personnel, and response rate to the survey at Mutualia (2009 and 2017).

	Mutualia 2009	Mutualia 2017
Number of hospitals	2	3
Number of professionals	349	395
Number of responses	130	152
Response rate (%)	37.2	38.5
Tenure in current profession (years, mean ± SD)	14.4 ± 9.6	18.1 ± 9.43 **
Tenure with Mutualia (years, mean ± SD)	11.1 ± 9.3	13.5 ± 8.9 *
Tenure in current work area (years; median and range)	6 (1–35)	11 (40–1) **
Full-time employees (>37.5 h/s) (%)	89.54	90.5 (n.s.)

Asterisks indicate significant differences between 2009 and 2017 data: n.s.: *p* > 0.05; *: *p* < 0.05; **: *p* < 0.01.

**Table 2:** Distribution by hospital service (work area) of the personnel who responded to the survey at Mutualia (2009 and 2017).

	Mutualia 2009	Mutualia 2017
1. Anesthesiology/Reanimation		
2. Surgery		
3. Diverse Units	3.3%	10.7%
4. Pharmacy		
5. Laboratory		
6. Internal Medicine	1.7%	0.7%
7. Obstetrician and Gynecology		
8. Admission	5.8%	8.1%
9. Nephrology		
10. Urology		
11. Oncology		
12. Pediatrics		
13. Radiology	2.5%	4.7%
14. Rehabilitation	33.1%	20.1%
15. Mental Health/Psychiatry		
16. Emergency Service	14.0%	14.1%
17. ICU (any type)		
18. Neurology		
19. Traumatology	27.3%	18.1%
20. Hemodialysis		
21. Nuclear Medicine		
22. Other	12.4%	18.1%

**Table 3:** Distribution by current job post of the personnel who responded to the survey at Mutualia (2009 and 2017).

Job Post	Mutualia 2009	Mutualia 2017
Management		
Administration	8.2%	14.2%
Nursing Assistant	11.5%	7.7%
Dietician		
Nurse	21.3%	32.4%
Pharmacist		
Resident Pharmacist		
Physiotherapy	22.1%	14.9%
Doctor	29.5%	24.3%
Resident Doctor	0.8%	0.7%
Technician (ECG, laboratory, X-ray diagnosis…)	2.5%	2.0%
Other personnel	4.1%	4.1%

**Table 4:** Scores obtained in each aspect at Mutualia (2009 and 2017).

Aspects of the Survey	Mutualia 2009		Mutualia 2017		*p* Value
	% Positive (Mean ± SD)	95% CI (%)	% Positive (Mean ± SD)	95% CI (%)	
1. Frequency of reports of events	53.51 ± 7.42	46.53–60.47	68.60 ± 5.04	63.86–73.30	*p* < 0.01
2. Perception of Safety	59.15 ± 13.8	48.50–69.85	65.12 ± 15.39	53.21–77.04	n.s.
3. Expectations and actions of management/supervisors of the unit/service which foster PS	73.19 ± 8.41	59.81–86.59	70.23 ± 9.05	55.85–84.65	n.s.
4. Organizational learning/continuous improvement	61.14 ± 19.87	42.48–79.79	69.38 ± 14.85	55.42–83.31	n.s.
5. Teamwork in the unit/service	79.13 ± 7.65	66.98–91.32	77.93 ± 7.14	66.56–89.29	n.s.
6. Frankness in communication	54.06 ± 12.61	48.50–72.17	59.29 ± 9.96	52.08–70.78	n.s.
7. Feedback and communication of errors	58.48 ± 5.35	51.45–61.49	68.94 ± 6.56	62.80–75.13	*p* < 0.05
8. Non-punitive response to errors	60.34 ± 10.92	51.94–58.73	61.45 ± 8.26	54.8–68.06	n.s.
9. Sufficient personnel	65 ± 10.94	47.59–82.41	54.28 ± 4.01	47.89–60.66	n.s.
10. Support of hospital management regarding patient safety	65.53 ± 3.15	57.75–73.4	73.89 ± 4.74	62.11–85.69	n.s.
11. Teamwork among the units/services	57.46 ± 9.01	50.47–64.43	64.59 ± 9.75	49.09–80.11	n.s.
12. Problems in shift changes and transitions between services	65.24 ± 7.29	53.64–76.86	69.44 ± 4.2%	62.7–76.15	n.s.

**Table 5:** Strengths and Weaknesses Table.

	Strengths			Weaknesses		
Dimension	Item/Question	2009	2017	Item/Question	2009	2017
Dimension 2				The pace of work is never increased if that would imply sacrificing patient safety (Q 15)	46.7%	46.1%
Dimension 3	My supervisor/boss overlooks patient safety problems which occur habitually (Q 22)	83.7%	80.3%			
Dimension 4	When a fault is detected in patient care, appropriate measures are taken to prevent it from happening again (Q 9)	83.1%	85.3%			
Dimension 5	Teamwork in the Unit/Service	79%	77.9%			
	Personnel support each other (Q 1)	82.3%	84.1%			
	In this unit, we treat each other with respect (Q 4)	88.5%	84.1%			
Dimension 6	When personnel see something that could negatively affect the care a patient receives, they can speak about it with complete freedom (Q 35)	71.5%	75%	Frankness in communication	59.3%	54%
				Personnel may question the decisions or actions of supervisors with complete freedom (Q 37)	35.3%	37%
Dimension 8				When an error is made, personnel fear that this may be reflected in their file (Q 16)	50.3%	48.5%
Dimension 9				Sufficient personnel	65%	54.3%
Dimension 10	The management or directors of the hospital provide a working climate that fosters patient safety (Q 23)	69.2%	75%			
	The management or directors of the hospital prove with actions that patient safety is one of their priorities (Q 30)	63.6%	78%			
Additional information	Information concerning patient’s diagnosis is communicated clearly and quickly to all the professionals involved in the care of that patient.	75.9%	80.9%			

## Data Availability

The data that support the findings of this study are available from the corresponding author, upon reasonable request.

## Appendix A

**Table A1:** Aspects of the safety culture and the items it includes. The corresponding question number on the questionnaire appears in parentheses.

Aspects of the Safety Culture	
Block A. Results of the safety culture	
1. Frequency of notified events	The errors that are discovered and corrected before affecting the patients are notified (Q 40). The errors that are not going to foreseeably harm the patient are notified (Q 41). The errors that have not had adverse consequences, even though they could have foreseeably harmed the patient, are notified (Q 42).
2. Perception of safety	The pace of work is never increased if that would imply sacrificing patient safety (Q 15). Our procedures and work resources are good for preventing errors in providing care (Q 18). More mistakes are not made by chance (Q 10). In this unit, there are problems related to patient safety (Q 17).
Block B. Aspect of the culture of safety at the unit/service level	
3. Expectations and actions of directors and supervisors of the Unit/Service which foster safety	My supervisor/boss expresses their satisfaction when we try to prevent risks to patient safety (Q 19). My supervisor/boss seriously takes into account the suggestions personnel make to improve patient safety (Q 20). When work pressure increases, my supervisor/boss tries to get us to work more quickly, even if that would put the patient’s safety at risk (Q 21). My supervisor/boss overlooks patient safety problems which occur habitually (Q 22).
4. Organizational learning/Continuous improvement	We have activities aimed at improving patient safety (Q 6). When a fault is detected in patient care, appropriate measures are taken to prevent it from happening again (Q 9). The changes we make to improve patient safety are evaluated so as to check their effectiveness (Q 13).
5. Teamwork in the Unit/Service	Personnel support each other (Q 1). When we have a lot of work, we all collaborate as a team to finish it (Q 3). In this unit, we treat each other with respect (Q 4). When someone is overloaded with work, they can get help from their colleagues (Q 11).
6. Frankness in communication	When personnel see something that could negatively affect the care a patient receives, they can speak about it with complete freedom (Q 35). Personnel may question the decisions or actions of supervisors with complete freedom (Q 37). Personnel fear asking questions about what seems to be something done incorrectly (Q 39).
7. Feedback and communication about errors	When we notify of an incident, we are informed about what type of actions have been carried out (Q 34). We are informed about errors that occur in this service/unit (Q 36). In my service/unit, we argue about how we can prevent an error from occurring again (Q 38).
8. Non-punitive response to errors	If colleagues or superiors discover you have committed an error, they use it against you (Q 8). When an error is detected, before trying to find the cause, they try to try to lay blame (Q 12). When an error is made, personnel fear that this may be reflected in their file (Q 16).
9. Sufficient personnel	There are enough personnel to deal with the workload (Q 2). Sometimes, the best patient care cannot be provided because the workday is exhausting (Q 5). Occasionally, the best patient care is not provided because there are too many substitutes or temporary personnel (Q 7). We work under pressure to carry out too many things in too much of a hurry (Q 14).
10. Hospital management support regarding patient safety	The management or directors of the hospital provide a working climate that fosters patient safety (Q 23). The management or directors of the hospital prove with actions that patient safety is one of their priorities (Q 30). The management or directors of the hospital only seem to be interested in patient safety when an adverse event happens to a patient (Q 31).
Block C. Aspects of the culture of safety at the whole hospital level	
11. Teamwork between units/services	There is close cooperation between the units/services which have to work together (Q 26). Services/units work together in a coordinated way to provide the best care possible (Q 32). Different hospital units are not well coordinated (Q 24). It is usually uncomfortable to work with personnel from other units/services (Q 28).
12. Problems when shifts change or in transitions between services/units	Patient information is lost, in part, when transferred from one unit/service to another (Q 25). During shift changes, important information about the care a patient has received is frequently lost (Q 27). The exchange of information between different services is regularly problematic (Q 29). Problems with patient care arise as a consequence of shift changes (Q 33).
